# Menstrual Cycle and Hormonal Contraceptives in Female Athletes: Should Symptoms and Nutrition Matter More than Cycle Phase? A Narrative Review

**DOI:** 10.3390/nu18071144

**Published:** 2026-04-02

**Authors:** Valentina Natalucci, Gioi Spinello, Tatiana Moro, Gaspare Pavei, Gennaro Boccia, Antonio La Torre, Matteo Bonato

**Affiliations:** 1 Department of Experimental and Clinical Medicine, Università Politecnica delle Marche, 60126 Ancona, Italy; 2 Department of Pathophysiology and Transplantation, University of Milan, 20133 Milan, Italy; gaspare.pavei@unimi.it; 3 Department of Biomedical Sciences, University of Padova, 35131 Padova, Italy; gioi.spinello@studenti.unipd.it (G.S.); tatiana.moro@unipd.it (T.M.); 4 Italian Athletics Federation (FIDAL), 00191 Rome, Italy; gennaro.boccia@unito.it (G.B.); antonio.latorre@unimi.it (A.L.T.); 5 Department of Clinical and Biological Sciences, University of Turin, 10126 Turin, Italy; 6 Department of Biomedical Sciences for Health, University of Milan, 20122 Milan, Italy; matteo.bonato@unimi.it; 7 Laboratory of Movement and Sport Sciences (LaMSS), IRCCS Istituto Ortopedico Galeazzi, 20157 Milan, Italy

**Keywords:** menstrual cycle, hormonal contraceptives, female athletes, athletic performance, metabolism, nutrition, neuromuscular function, injury risk, symptom monitoring

## Abstract

Background: Understanding how endogenous hormonal fluctuations and exogenous hormonal modulation influence exercise-related outcomes in women is essential for developing individualized and evidence-informed training and nutritional strategies. This narrative review summarizes the endocrine physiology of the eumenorrheic menstrual cycle and hormonal contraceptive (HC) use and critically examines their implications for athletic performance, neuromuscular function, injury risk, and metabolic regulation in physically active women. Methods: A non-systematic literature search was conducted in PubMed, Scopus, and Web of Science for articles published up to January 2026. Search terms combined menstrual cycle-related, hormonal contraceptive, performance, and metabolic/nutritional keywords, and relevant studies were selected based on their relevance to the scope of this narrative review. Results: Estradiol and progesterone fluctuations may modulate substrate utilization, connective tissue properties, central fatigue regulation, and symptom expression; however, evidence indicates that performance-related effects across menstrual phases are generally small and inconsistent, reflecting both the modest magnitude of physiological effects and the methodological heterogeneity in menstrual cycle phase classification and verification across studies. Similarly, although HC use suppresses endogenous hormonal variability, current findings do not support consistent benefits for performance, injury prevention, or metabolic outcomes, and responses remain heterogeneous. From a nutritional perspective, the endocrine context may contribute to modest changes in energy expenditure, insulin sensitivity, appetite regulation, inflammation, and recovery-related processes. Importantly, symptom burden—including pain, fatigue, sleep disturbances, gastrointestinal discomfort, and fluid retention—emerges as a practical driver of day-to-day training tolerance. Conclusions: We propose an integrative framework in which sex hormones define a physiological context rather than deterministic performance regulators, while nutrition acts as a key modifiable factor influencing metabolic responses, symptom severity, and performance consistency.

## 1. Introduction

Biological sex is widely recognized as an important determinant of performance in many physical activities and sporting disciplines, influencing multiple physiological systems involved in movement production and response to exercise [1]. While gender equality remains a fundamental social and ethical principle, it does not imply equivalence in physiological responses to training and competition, which are shaped by sex-specific biological and hormonal differences [2,3]. Historically, sport and exercise science research has largely relied on male-based models, resulting in knowledge gaps and oversimplified assumptions when applied to female athletes [2].

In the present review, the term “physically active and athletic women” refers broadly to women engaged in regular physical activity and sport participation, including recreationally active individuals as well as trained and competitive athletes.

In women, sex-specific physiology is further characterized by dynamic endocrine modulation across the lifespan. Unlike the relatively stable hormonal environment typically observed in men, postpubescent women experience cyclical fluctuations in ovarian hormones throughout the menstrual cycle [4], as well as distinct endocrine profiles associated with hormonal contraceptive (HC) use [5]. These fluctuations primarily involve estradiol and progesterone, hormones that exert pleiotropic effects extending well beyond reproductive function. Both hormones influence metabolic regulation—including substrate utilization, insulin sensitivity, and protein turnover [6]—and may also modulate neuromuscular activation, muscle–tendon and connective tissue properties, and central nervous system processes [7]. Collectively, these endocrine-mediated interactions have important implications for athletic performance, training adaptation, recovery, and injury risk. Consequently, understanding how endogenous hormonal variability and exogenous hormonal modulation interact with these physiological systems is essential for developing evidence-informed and individualized strategies for female athletes.

Despite extensive research on menstrual cycle phases and HC use, their effects on performance-related physiological outcomes are generally small, highly variable, and difficult to translate into practical recommendations. Notably, even when menstrual cycle phases are verified using direct hormonal measurements, substantial overlap in endocrine profiles and functional responses persists, limiting the predictive value of phase-based classifications alone [8,9,10].

From a nutritional and metabolic perspective, endocrine fluctuations across the menstrual cycle may be associated with phase-dependent variations in energy expenditure, appetite regulation, substrate utilization, glycemic control, inflammation, and recovery-related processes. Likewise, exogenous hormonal modulation through HC use may influence metabolic responses and nutrient requirements, although current findings remain formulation-dependent and highly individualized. Importantly, nutritional status does not merely represent an outcome influenced by hormonal context but may act as a key modulator of physiological responses, symptom expression, and training tolerance. In this regard, energy availability represents a central integrative construct linking dietary intake, exercise energy expenditure, endocrine function, and downstream outcomes relevant to performance and health. Accordingly, when a state of energy imbalance is prolonged, low energy availability may occur with direct consequences on endocrine regulation and performance. Low energy availability is recognized as the primary etiological factor underlying relative energy deficiency in sport, a syndrome that encompasses multi-system physiological impairments resulting from insufficient energy to support athletic demands and essential physiological functions. Low energy availability links directly to alterations in hormonal status, manifesting in women as menstrual disturbances and other endocrine disruptions. These alterations may ultimately impair performance outcomes by compromising training tolerance, recovery process, metabolic efficiency, and increasing symptoms that interfere with training and performance [11].

Moreover, emerging evidence suggests that subjective and objective symptoms (e.g., pain, fatigue, sleep disturbances, gastrointestinal discomfort, and fluid retention) may exert a stronger influence on day-to-day performance and training availability than the hormonal phase itself [12,13,14]. Such symptoms may reflect the integrated effects of endocrine modulation on central regulation, metabolic function, and behavioral responses and are increasingly recognized as practical targets for individualized nutritional and training interventions.

Therefore, the purpose of this narrative review is to summarize current knowledge on the endocrine physiology of the eumenorrheic menstrual cycle (28-day) and HC use and to critically examine their implications for athletic performance, neuromuscular function, injury risk, and metabolic and nutritional outcomes. By integrating mechanistic evidence with applied considerations, this review proposes a conceptual framework in which hormonal fluctuations define the physiological context, while interindividual variability and symptom expression shape functional outcomes. Within this framework, nutrition emerges as a key modifiable factor influencing energy availability, training tolerance, recovery capacity, and performance consistency, supporting individualized, symptom-informed strategies for physically active and athletic women.

## 2. Methods

To identify relevant literature on the interactions between menstrual cycle physiology, HC use, and exercise-related outcomes in women, a structured literature search was conducted in major scientific databases, including PubMed, Scopus, and Web of Science. Search terms combined menstrual cycle-related keywords (e.g., menstrual cycle, follicular, luteal, ovulation, estradiol, progesterone) and HC terms (e.g., hormonal contraceptives, oral contraceptives, progestin-only) with exercise/performance outcomes (e.g., strength, endurance, fatigue, running economy) and metabolic/nutrition-related terms (e.g., substrate oxidation, glycemic control, energy availability, appetite, hydration, iron/hepcidin). The search covered studies published up to January 2026; earlier relevant studies were included when necessary to provide a physiological context, and recently published articles identified during manuscript revision were incorporated when relevant. The search returned approximately 700 records across databases (excluding duplicates). Titles and abstracts were screened to identify studies relevant to the scope of this review. Priority was given to peer-reviewed original research articles, systematic reviews, and meta-analyses published in English. Additional relevant publications were identified through manual screening of the reference lists of key articles.

Given the narrative nature of this review, studies were selected based on their relevance to the conceptual framework and applied topics addressed in the manuscript rather than through predefined systematic inclusion and exclusion criteria. We included studies using gold-standard hormonal verification methods (e.g., serum estradiol and progesterone measurements combined with luteinizing hormone surge detection) and studies relying on calendar-based or self-reported cycle tracking. Although this approach may introduce phase misclassification and contribute to variability, it encompasses a broader body of literature, given that gold-standard verification remains underutilized in studies assessing performance outcomes.

## 3. Endocrine Context: Natural (Eumenorrheic) Menstrual Cycle and Hormonal Contraceptive Use

Sex-specific endocrine regulation represents a fundamental component of female physiology and provides the biological context through which exercise-related responses may vary across individuals. In women, this context is shaped by both endogenous hormonal fluctuations across the natural (eumenorrheic) menstrual cycle and exogenous hormonal modulation induced by HC use. Importantly, these endocrine environments do not act as direct determinants of performance; rather, they interact with metabolic regulation and nutrition-related factors, as well as neuromuscular and central regulatory systems, contributing to substantial interindividual variability in functional outcomes [Table 1].

### 3.1. Natural (Eumenorrheic) Menstrual Cycle

#### 3.1.1. Hormonal Fluctuations Across the Cycle

The natural (eumenorrheic) menstrual cycle comprises two coordinated but distinct biological processes: the ovarian cycle and the uterine (endometrial) cycle. The ovarian cycle includes the follicular phase, ovulation, and the luteal phase, whereas the endometrial cycle consists of the menstrual, proliferative, and secretory phases, followed by a premenstrual period. Overall, the ovarian follicular phase overlaps with the menstrual and proliferative phases of the endometrium, while the ovarian luteal phase largely corresponds to the secretory phase [15].

Although the menstrual cycle is often described using a 28-day model for illustrative purposes, considerable physiological variability exists. In eumenorrheic women, cycle length typically ranges from approximately 21 to 35 days, and variability in ovulation timing, luteal phase characteristics, or the occurrence of luteal phase defects may further modify the endocrine context.

**Figure 1:**
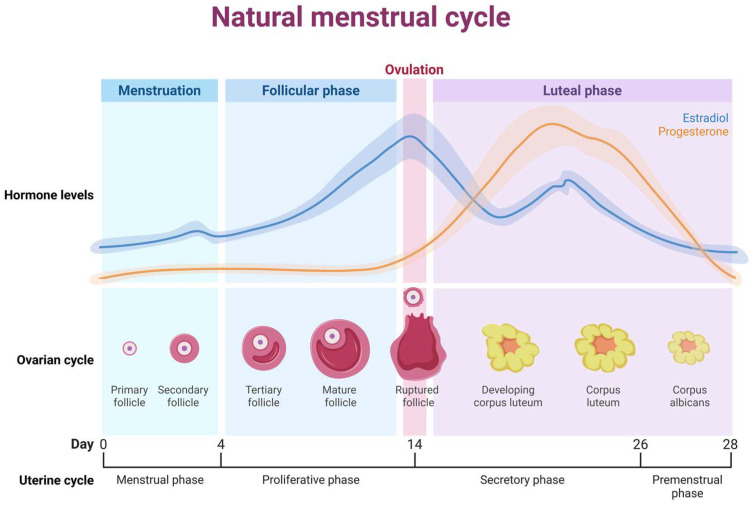
Schematic representation of a typical 28-day cycle and associated estradiol and progesterone fluctuations.

From an endocrine perspective, the cycle is characterized by cyclical fluctuations in ovarian hormones, primarily estradiol and progesterone. During the early follicular phase, circulating concentrations of both hormones are relatively low. Estradiol progressively increases throughout the mid-to-late follicular phase, reaching a peak around ovulation, while progesterone remains low. Following ovulation, progesterone rises markedly during the luteal phase, accompanied by moderately elevated estradiol levels, before both hormones decline during the late luteal phase preceding menstruation [Figure 1] [15].

Although these hormonal patterns are often described as predictable, substantial interindividual variability exists in absolute hormone concentrations, timing of hormonal peaks, and hormonal sensitivity. Consequently, menstrual cycle phases should be viewed as conceptual frameworks rather than uniform physiological states, particularly when interpreting exercise-related outcomes [16,17]. Moreover, in sport and exercise research, menstrual cycle phase classification is frequently based on calendar methods or self-reported data rather than direct hormonal verification, which may further contribute to variability and inconsistencies across studies. Even when cycle phases are verified using direct serum measurements of estradiol and progesterone, considerable overlap in hormonal concentrations and functional responses persists, limiting the predictive value of phase-based classifications alone [8,10,16]. From an applied perspective, this overlap also cautions against rigid phase-based nutritional assumptions and generalized recommendations.

#### 3.1.2. Systemic Effects of Estradiol and Progesterone

Estradiol and progesterone exert pleiotropic effects across multiple physiological systems relevant to exercise and recovery, acting as systemic modulators rather than hormones with exclusively reproductive functions. Through interactions with peripheral tissues and the central nervous system, fluctuations in these hormones influence metabolic regulation, neuromuscular function, connective tissue properties, segmental volumes, and perceptual responses [18].

From a metabolic perspective, estradiol has been associated with enhanced lipid oxidation, improved insulin sensitivity, and modulation of carbohydrate utilization, potentially supporting more efficient substrate use during exercise and recovery [19,20]. Estradiol may also contribute to muscle-protective effects through influences on protein turnover and mitochondrial function. In contrast, progesterone is associated with increased basal body temperature, altered ventilatory responses, and potential antagonistic effects on estradiol-mediated metabolic actions, including less favorable glycemic regulation under certain conditions [19]. Collectively, these endocrine mechanisms provide a physiological rationale for nutrition-related considerations across the cycle, particularly regarding carbohydrate handling, recovery nutrition, and hydration needs.

At the musculoskeletal level, estradiol has been linked to modulation of connective tissue metabolism and muscle–tendon mechanical properties, with potential implications for tissue stiffness, load tolerance, and recovery [21,22]. However, causal relationships between hormonal fluctuations and injury risk remain difficult to establish, given the multifactorial nature of musculoskeletal loading, adaptation, training load, fatigue accumulation, and sport-specific injury mechanisms.

Estradiol and progesterone also interact with the central regulatory system, influencing pain perception, fatigue, thermoregulation, mood, and sleep regulation [23,24,25,26,27]. These central effects may indirectly influence exercise tolerance and recovery quality, thereby contributing to day-to-day variability in training readiness and perceived performance. Importantly, the presence of plausible endocrine mechanisms does not necessarily translate into consistent or predictable functional effects at the individual level [10].

#### 3.1.3. Interindividual Variability and Functional Relevance

Despite well-described hormonal patterns across the menstrual cycle, functional responses to exercise vary markedly between individuals. Differences in hormonal sensitivity, receptor expression, symptom burden, training background, nutritional availability, recovery capacity, and psychosocial factors contribute to heterogeneous physiological, perceptual, and performance-related responses [8,9]. In trained women, high training loads and elevated energy expenditure may disrupt hormonal balance by inducing low energy availability, suppressing reproductive hormone production, and contributing to menstrual disturbances [28,29]. Such disruptions may manifest as reduced estradiol and progesterone concentrations, luteal phase defects, anovulatory cycles, or irregular bleeding patterns, ultimately limiting the interpretability of standard phase-based classifications in the athletic population [28].

Accumulating evidence indicates that interindividual variability often exceeds average phase-related effects, thereby limiting the explanatory and predictive value of menstrual cycle phase alone [30,31]. Consequently, hormonal fluctuations should be interpreted as shaping a physiological context that interacts with training- and nutrition-related factors, rather than determining uniform functional outcomes. This perspective reinforces the need for individualized, symptom-informed, and nutrition-aware approaches rather than reliance on rigid phase-based assumptions in applied settings.

### 3.2. Hormonal Contraceptive Use

#### 3.2.1. Types and Mechanisms of Action

HCs encompass a wide range of formulations, including combined estrogen-progestin contraceptives and progestin-only methods, delivered via oral, transdermal, vaginal, injectable, or long-acting routes [32,33]. Despite differences in formulation and delivery, their primary mechanism of action includes suppression or inhibition of ovulation through modulation of the hypothalamic–pituitary–ovarian axis, together with changes in cervical mucus and endometrial environment that reduce the likelihood of fertilization and implantation.

#### 3.2.2. Endocrine Environment Induced by Hormonal Contraceptives

The endocrine milieu induced by HC use does not replicate the hormonal profile of any specific phase of the natural menstrual cycle. Instead, it reflects chronic exposure to synthetic hormones combined with attenuated endogenous ovarian hormone production. Synthetic estrogens and progestins differ from endogenous hormones in their pharmacokinetics, receptor-binding properties, and downstream signaling effects, thereby creating a distinct and non-physiological endocrine environment [34,35]. Importantly, synthetic progestins also differ in their androgenicity and receptor-binding profiles, which may lead to distinct metabolic, cardiovascular, and neuroendocrine effects depending on the formulation used. Consequently, different HC formulations may influence metabolic regulation, neuromuscular function, and substrate utilization during exercise. In particular, suppression of endogenous estradiol combined with exposure to synthetic hormones may modify estrogen receptor signaling and downstream metabolic pathways, potentially influencing lipid oxidation, carbohydrate metabolism, and skeletal muscle responses to exercise.

#### 3.2.3. Implications for Metabolic Regulation and Symptom Expression

HC use may influence metabolic responses, including substrate utilization, insulin sensitivity, and appetite regulation, although reported effects are generally modest, heterogeneous, and likely influenced by individual characteristics and contraceptive type [36,37]. Similarly, symptom expressions, such as fatigue, mood changes, gastrointestinal discomfort, or fluid retention, vary widely among users [38].

Overall, assumptions that HCs uniformly stabilize physiological or performance-related responses are not supported by current evidence. Rather than reducing variability, HCs introduce a different endocrine context within which individual metabolic and symptomatic responses emerge [39]. This reinforces the need to consider HC users as a distinct physiological population and to prioritize individualized monitoring strategies rather than generalized assumptions of hormonal “stabilization”.

## 4. Physiological Systems Potentially Sensitive to Endocrine Context

**Figure 2:**
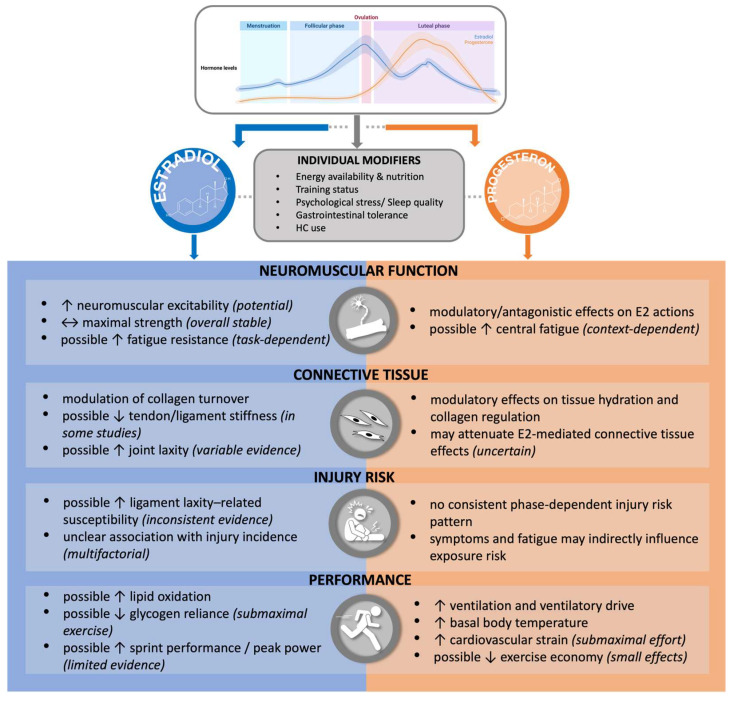
Conceptual framework illustrating the endocrine context of the eumenorrheic menstrual cycle and its potential modulatory effects on key physiological systems relevant to athletic performance. **Abbreviations:** E2, estradiol; HC, hormonal contraceptive. **Symbols:** ↑ increase; ↓ decrease; ↔ no consistent change. Arrows represent the potential direction of endocrine modulation and should not be interpreted as deterministic effects.

Endogenous hormonal fluctuations across the menstrual cycle and the distinct endocrine milieu induced by HC use may influence multiple physiological systems relevant to athletic performance. Rather than acting through a single pathway, endocrine context may shape performance through integrated effects on neuromuscular function, muscle–tendon behavior, connective tissue properties, and central regulatory processes. Across these domains, reported effects are typically modest at the group level and strongly influenced by task demands, training status, symptom burden, and interindividual variability, which together limit the predictive value of endocrine status alone [Figure 2].

### 4.1. Neuromuscular Function and Muscle–Tendon Behavior

Neuromuscular function and muscle–tendon behavior are important determinants of athletic performance and may be influenced by endocrine context through changes in neural drive and tissue mechanics. Estradiol has been linked to increased corticospinal excitability, possibly through intrinsic changes in descending pathways [40], whereas progesterone may modulate or oppose these effects under some conditions [10,40]. However, evidence that these mechanisms translate into consistent functional changes remains mixed.

At the muscle–tendon level, studies in non-athletic women suggest that estradiol may affect connective tissue turnover and viscoelastic properties, with potential effects on tendon stiffness, compliance, and force transmission [21,41,42]. Estradiol has also been associated with reduced collagen synthesis and altered fibroblast activity, while progesterone may partly attenuate these effects [41,42]. These mechanisms may contribute to cycle-related changes in joint laxity, stability, and tissue loading reported in some studies [43,44].

Nevertheless, mechanistic findings do not consistently match functional outcomes. Cycle-related differences have been reported in neuromuscular control [45], proprioception [46], and muscle stiffness [22], whereas studies measuring tendon stiffness often report no significant variation across menstrual cycle phases [47,48,49]. Overall, endocrine context may influence neuromuscular and muscle–tendon function, but effects appear small, inconsistent, and strongly shaped by interindividual variability and contextual factors such as fatigue, task demands, and symptoms.

### 4.2. Injury Risk and Musculoskeletal Health

Sports injuries arise from multifactorial interactions among anatomical structure, biomechanics, tissue capacity, training load, and environmental factors. In this context, endocrine fluctuations across the menstrual cycle and HC use have been investigated as potential contributors to musculoskeletal injury risk in female athletes, primarily through their association with joint laxity, movement control, fatigue, and symptom-related constraints on training and recovery.

Female athletes have a higher incidence of some injuries than males, especially knee ligament injuries, which occur about two to five times more often in women participating in the same sports [50]. As a result, anterior cruciate ligament (ACL) injury has been studied extensively because of its anatomical, biomechanical, and physiological determinants [51]. Several studies have examined whether injury incidence varies across menstrual cycle phases, but findings remain inconsistent. A systematic review by Martinez-Fortuny et al. [52] suggested greater injury susceptibility during ovulation, supported by evidence of increased ligamentous laxity [53]. Other studies have reported higher injury incidence during the late follicular phase [54,55]. These associations should be interpreted cautiously because injury risk is also influenced by training load, accumulated fatigue, competition schedule, and sport-specific biomechanical demands.

Importantly, cycle-related symptoms may represent an additional and potentially more proximal contributor to injury susceptibility than the hormonal phase alone. In young elite female athletes, greater fatigue and poorer sleep quality during the luteal phase were associated with an increased incidence of joint, ligament, and muscle injuries [56], supporting the concept that symptom burden and functional readiness may influence exposure and movement quality.

HC use may further modify injury-related profiles by suppressing ovulation and attenuating endogenous hormonal peaks. Although some studies have suggested a potential protective association between HC use and musculoskeletal injury risk [57,58,59], a recent systematic review reported inconclusive associations between HC use and injury outcomes [60]. Moreover, potential adverse effects, including concerns related to bone health and ovulatory function, remain relevant, and long-term implications for musculoskeletal outcomes are uncertain [61]. Overall, current evidence is insufficient to recommend HC use as an injury-prevention strategy, reinforcing the need to prioritize multifactorial risk management and individualized monitoring.

### 4.3. Athletic Performance Outcomes

A recent systematic review using rigorous menstrual cycle phase verification, including serum estradiol and progesterone assessment combined with luteinizing hormone surge detection, provides one of the strongest evaluations of cycle-related effects on athletic and neuromuscular performance to date [10]. When analyses are restricted to studies with precise hormonal classification, maximal and explosive strength appear largely unaffected by menstrual cycle phase at the group level, whereas neuromuscular coordination and some performance-related outcomes show clearer phase-dependent variation.

### 4.4. Endurance Performance and Submaximal Physiology

Mechanistically, estradiol has been associated with enhanced lipid oxidation and reduced glycogen reliance [62], while progesterone during the mid-luteal phase has been linked to increased ventilatory drive, elevated basal body temperature, and greater cardiovascular strain [63]. Consistent with these mechanisms, maximal aerobic performance (e.g., $\dot{V}$O_2max_, time to exhaustion, time-trial performance) appears largely stable across the menstrual cycle phases [64]. In contrast, submaximal endurance physiology shows clearer modulation, including changes in ventilation, carbohydrate oxidation, and running economy, particularly during the mid-luteal phase, likely reflecting progesterone-mediated effects [65,66,67,68]. These effects do not appear to impair performance consistently, but they may matter in high-performance settings where small changes in economy can affect competition outcomes [69].

### 4.5. Strength and Power

Earlier reviews suggested that the early follicular phase might impair maximal strength [70], but these conclusions were based largely on studies with limited phase-verification rigor. In studies using stricter hormonal verification, maximal isometric and isokinetic strength appear largely unaffected by cycle phase [10]. Similarly, most studies report no consistent effects on explosive strength or jump performance [55,71,72]. Some isolated findings describe lower peak power or countermovement jump height during the early follicular phase and associations between performance and psychological variables such as motivation, perceived readiness, pleasure, and arousal [73]. Overall, average effects on strength and power appear minimal, although responses vary meaningfully between individuals.

### 4.6. Sprint/Anaerobic Performance and Coordination

Anaerobic cycling sprint performance may be more sensitive in some studies, with lower peak power during low-hormone phases and more favorable responses during the late follicular and mid-luteal phases [73,74]. Reduced fatigability during repeated sprinting in the late follicular phase has also been reported [73], possibly reflecting estradiol-related neuroexcitatory effects and improved adenosine triphosphate (ATP) availability [75]. Evidence on neuromuscular coordination and agility is limited, but some studies suggest better performance around ovulation and poorer performance during the early follicular phase [72,76,77]. Although these findings are compatible with estradiol-mediated effects on neural function when progesterone is low [75], the evidence remains limited and heterogeneous.

### 4.7. Sport-Specific Performance

Findings for sport-specific performance are also mixed. Sub-elite female soccer players showed reduced total distance covered and fewer sprint efforts across the early follicular, late follicular, and mid-luteal phases [78], whereas elite players showed no significant differences between follicular and luteal phases [79]. These observations suggest that competitive level, training background, and fatigue resistance may influence whether endocrine context translates into measurable sport-performance differences.

## 5. Metabolic and Nutritional Considerations

Metabolic regulation represents a key interface between endocrine context, training load, and functional performance in physically active and athletic women. Fluctuations in sex hormones across the menstrual cycle, as well as exogenous hormonal exposure induced by HC use, may influence energy expenditure, substrate utilization, appetite regulation, insulin sensitivity, inflammation, and recovery-related processes. Importantly, nutrition should not be viewed solely as an outcome of endocrine modulation, but rather as an active factor capable of shaping metabolic responses, symptom expression, and training tolerance.

### 5.1. Energy Expenditure, Substrate Utilization, and Insulin Sensitivity

Cyclical hormonal fluctuations across the menstrual cycle have been associated with modest changes in energy expenditure and substrate utilization, although findings remain heterogeneous. Progesterone-related increases in basal body temperature during the luteal phase may contribute to small elevations in energy expenditure [80], whereas estradiol has been linked to enhanced lipid oxidation and fatty acid availability, favoring lipid over carbohydrate oxidation primarily during prolonged exercise at low-to-moderate intensities (typically between 45% and 65% VO_2_peak) and through increased skeletal muscle fatty acid utilization at rest [19,81].

While these metabolic shifts are generally small at the group level, they may become relevant under conditions of high training load, reduced energy availability, or limited recovery capacity.

Central insulin signaling plays a key role in integrating endocrine and metabolic regulation. During the follicular phase, cerebral insulin action appears to exert favorable effects on systemic insulin sensitivity, whereas this effect is attenuated or absent during the luteal phase, suggesting a phase-dependent reduction in central insulin sensitivity [80]. Such alterations may contribute to glycemic variability and appetite-related changes observed in some women during the luteal phase [82].

From an applied nutritional perspective, these findings support the importance of maintaining adequate carbohydrate availability and overall energy intake across the cycle, while recognizing that interindividual variability in metabolic flexibility and symptom expression may exceed average phase-related effects.

### 5.2. Appetite Regulation, Cravings, and Energy Intake

Appetite fluctuations across the menstrual cycle arise from complex interactions between hormonal changes, central metabolic regulation, and inflammatory signaling, influencing both energy intake and food preference. The most pronounced changes in appetite and cravings are consistently reported during the luteal phase, particularly among women experiencing premenstrual syndrome.

Women with premenstrual syndrome demonstrate significantly higher daily caloric intake (≈2200 kcal/day) compared with asymptomatic women (≈1880 kcal/day), alongside increased hedonic hunger, reflected by higher scores on the Power of Food Scale [83]. This increase may partly reflect physiological adaptations to higher energy expenditure, as resting energy expenditure can rise by approximately 100–300 kcal/day during the luteal phase, suggesting that modest increases in caloric intake may represent a compensatory response rather than maladaptive eating behavior [84]. This phase is also characterized by increased fat intake, potentially reflecting shifts in substrate preference and appetite regulation across the cycle [81].

Craving, defined as an intense desire for specific foods, has been associated with systemic inflammatory activity across the menstrual cycle. Elevated levels of high-sensitivity *C*-reactive protein, interleukin-6, and RANTES have been linked to an increased likelihood of moderate-to-severe cravings for chocolate, sweets, and savory foods, supporting the hypothesis that inflammatory processes may contribute to premenstrual eating behavior [85].

In contrast, evidence regarding peripheral appetite-regulating hormones remains inconsistent. In physically active women, no significant differences have been observed between early follicular and mid-luteal phases in acylated ghrelin, peptide YY (PYY), or subjective appetite ratings, even following intense exercise [86]. Minor phase-related effects on postprandial ghrelin (known as the “hunger hormone”) and PYY responses have been reported, but these do not appear to translate into clinically meaningful differences in hunger or satiety perception. Furthermore, PYY responses may be more strongly influenced by exercise intensity, gut environment, and immune-related interactions than by menstrual cycle phase itself [87,88].

From a nutritional perspective, practical strategies to stabilize appetite may include avoiding prolonged energy deficits, such as skipping meals, a behavior reported by up to 40% of athletes, which can disrupt hunger–satiety signaling and exacerbate fatigue and cravings [89]. Emphasis on nutrient-dense foods and whole grains while limiting highly processed foods and refined sugars may also help stabilize glycemic responses and reduce PMS symptom severity [90]. Distributing protein intake throughout the day (≈0.3 g·kg^−1^ every ~3 h) may further support satiety and postprandial energy expenditure [91], although this thermogenic effect may be attenuated in oral contraceptive users [92].

Collectively, these findings suggest that appetite and craving variability may be better explained by symptom burden and inflammatory modulation than by menstrual phase classification alone, highlighting the importance of individualized nutritional strategies in female athletes.

### 5.3. Muscle Protein Metabolism and Recovery

The impact of menstrual cycle phase and HC use on muscle protein metabolism and recovery has received increasing attention. Historically, the follicular phase has been proposed as a more favorable hormonal environment for anabolism due to estradiol peaks, whereas the luteal phase has been hypothesized to promote catabolism through progesterone-related mechanisms [93,94]. However, recent investigations using stable isotope tracer methodologies challenge this paradigm [95,96].

Integrated myofibrillar protein synthesis rates, both at rest and following resistance exercise, do not appear to differ significantly between the late follicular and mid-luteal phases [95,96]. These findings indicate that skeletal muscle anabolic responsiveness is largely preserved throughout the menstrual cycle, suggesting that the capacity to adapt to mechanical stimuli remains stable across phases [95]. Although progesterone has been associated with increased amino acid oxidation and may partially antagonize estrogen-mediated protective effects [94,97], systemic myofibrillar proteolysis does not appear to differ significantly between phases [95].

Notably, some athletes may report greater delayed onset muscle soreness during the early follicular phase, when circulating estradiol and progesterone are at their lowest concentrations [55]. While this finding does not necessarily indicate impaired anabolic adaptation, it may influence training tolerance and perceived recovery.

With respect to HCs, emerging evidence indicates that their use does not impair skeletal muscle anabolic responsiveness. Women using HCs have demonstrated greater gains in upper limb lean mass (5.5% vs. 2.9%) and vastus lateralis cross-sectional area (10.0% vs. 5.3%) compared with non-users following resistance training, without differences in strength expression [98]. However, systematic reviews suggest that overall HC use does not consistently influence global hypertrophy or physical performance outcomes [96].

Stable isotope tracer studies further show that the HC phase (active vs. inactive pill) does not significantly affect myofibrillar protein synthesis or proteolysis at rest or after exercise [99]. Together, these findings suggest that resistance training adaptations are not meaningfully dependent on menstrual phase or HC cycle week and do not support routine phase-specific manipulation of protein intake or training periodization in most athletes [96]. Although previous guidelines suggested increasing protein intake during the luteal phase (~12%) to counteract progesterone-related catabolism [84,91], recent evidence indicates that protein intake recommendations for female athletes should prioritize total daily intake (1.4–2.2 g/kg/die) and ensuring appropriate post-exercise protein intake (0.32–0.38 g/kg) to support recovery and adaptation rather than menstrual cycle phase adjustments [96].

### 5.4. Inflammation, Fluid Balance, and Gastrointestinal Function

Hormonal fluctuations across the menstrual cycle may influence inflammatory activity, fluid retention, and gastrointestinal function, thereby contributing to variability in training tolerance and perceived recovery. Elevated inflammatory markers during the luteal phase have been associated with increased fatigue, discomfort, and food cravings, reinforcing the link between inflammation and symptom burden [85].

Progesterone-related activation of the renin–angiotensin–aldosterone system may contribute to fluid retention and transient body mass fluctuations, while gastrointestinal symptoms such as bloating, abdominal discomfort, and altered bowel habits are frequently reported across specific cycle phases [100]. These responses are highly individual and may be exacerbated by inadequate hydration practices, electrolyte imbalance, or suboptimal dietary fiber intake [101].

From an applied perspective, nutritional strategies targeting hydration status, sodium balance, omega-3 fatty acid intake, and dietary fiber quality may support symptom management. However, given the wide interindividual variability in gastrointestinal symptoms and fluid retention, generalized phase-based recommendations should be applied cautiously and prioritized only when consistent symptom patterns are documented.

### 5.5. Metabolic Effects of Hormonal Contraceptives

HC use induces a distinct endocrine and metabolic environment that differs substantially from the eumenorrheic menstrual cycle. Combined HCs, particularly formulations containing androgenic progestins, have been associated with altered glucose metabolism, including impaired glucose tolerance and exaggerated postprandial glycemic and insulinemic responses during the active pill phase [102,103]. These effects appear to reflect changes in insulin sensitivity and glucose handling rather than uniform metabolic dysfunction and show considerable interindividual variability. Consequently, prioritizing adequate carbohydrate availability is essential during active weeks to compensate for the hormone-mediated suppression of gluconeogenesis [91], and prioritizing low-glycemic index carbohydrates may be a prudent strategy to counteract this transient insulin resistance.

HC use has also been linked to altered energy metabolism, including attenuation of diet-induced thermogenesis, suggesting modified macronutrient handling under exogenous hormonal exposure [104]. Such findings may have implications for body mass regulation and appetite control, particularly in athletes adopting high-protein dietary strategies. Regarding protein metabolism, while myofibrillar protein synthesis remains stable between active and inactive phases [99], HC use appears to eliminate the enhanced thermic effect of high-protein meals [92], which may necessitate more precise energy intake management. To support recovery, current guidelines suggest a daily protein target of 1.4–2.2 g·kg^−1^, including 0.32–0.38 g·kg^−1^ of high-quality protein post-exercise [91].

With respect to micronutrient status, HC users have been reported to exhibit reduced plasma concentrations of vitamin B6, a nutrient involved in neurotransmitter synthesis and central nervous system function [105]. A daily supplementation of 100 mg of vitamin B6 has been shown to reduce depressive symptoms by 20% in young women using combined oral contraceptives, which might be a proper dosage to overcome vitamin B6 deficiency [92,106]. Conversely, combined oral contraceptive use has been associated with higher circulating 25-hydroxyvitamin D concentrations, with increases of up to 35% reported [107]. This elevation appears to occur alongside normal physiological adjustments in calcium homeostasis and bone health [107], and oral contraceptive use has also been associated with 11.3% greater calcium retention in bone, suggesting a potential modulatory effect of oral hormonal contraception on bone metabolism [108]. However, vitamin D supplementation remains indicated in cases of deficiency (25(OH)D < 30 ng/mL) or in athletes with specific risk factors such as indoor training, high latitude or winter exposure, or darker skin pigmentation [109,110]. In these situations, 1000–2000 IU/day may support immune and musculoskeletal health [111]. Overall, supplementation should be guided by individual biochemical monitoring rather than contraceptive use alone, as combined oral contraceptive use may already be associated with higher circulating vitamin D levels [107]. Optimal levels for athletes are generally proposed within the range of 80–100 nmol/L (approximately 32–40 ng/mL) to support muscle function and reduce injury risk [109,112]. Adequate calcium intake is equally critical, with a recommended dietary intake of 1000 mg/day for adults, increasing to 1300–1500 mg/day in athletes to compensate for sweat losses and support bone mineral density [109,110].

Iron metabolism represents a particularly relevant topic in female athletes. Iron deficiency is reported to be approximately five- to seven-fold more prevalent in female athletes than in male counterparts, largely due to menstrual blood losses and hormonally mediated regulation of hepcidin. In eumenorrheic athletes, the early follicular phase may represent a favorable window for iron absorption, as low circulating sex hormones and the gradual rise in estrogen have been associated with reduced hepcidin activity, facilitating dietary iron uptake and utilization [111]. Conversely, during the luteal phase, elevated progesterone concentrations may be associated with increased hepcidin expression, potentially limiting iron absorption and recycling. This effect may be amplified by premenstrual low-grade inflammation, thereby contributing to functional iron deficiency [113].

From a clinical and applied perspective, iron deficiency exists along a continuum from non-anemic stages to overt anemia. Iron deficiency anemia is defined by hemoglobin concentrations < 120 g/L in female athletes. When hemoglobin is normal, iron deficiency can still be present and is classified as non-anemic: Stage 1 (ferritin < 30 µg/L, TSAT > 16%) and Stage 2 (ferritin < 20 µg/L, TSAT < 16%). These thresholds provide practical guidance, with ferritin < 30 µg/L commonly used as a trigger for intervention, although higher targets (e.g., 30–50 µg/L) may be appropriate in athletes with increased iron demands [112]. For example, the iron requirements of endurance athletes may be substantially higher, with estimates suggesting needs up to 70% greater (~30 mg/day) than those of the general population [109,112]. Strategies to optimize absorption may include co-ingestion with vitamin *C*-rich sources (e.g., orange juice), which can increase iron bioavailability by approximately fourfold [112].

In contrast, HC use may exert a protective effect against iron deficiency, primarily through reduced menstrual blood loss. Iron-containing HCs have been explored as a strategy to mitigate iron deficiency and anemia, although evidence supporting their superiority over standard formulations remains limited. Therefore, routine iron supplementation should not be recommended in HC users in the absence of biochemical evidence or medical indication, reinforcing the importance of individualized assessment.

Although HC use is often associated with reduced severity of menstrual-related symptoms and improved perceived well-being [114,115], potential metabolic challenges, including altered glucose regulation and inflammatory responses, highlight the need for individualized nutritional monitoring and tailored dietary strategies in women using HCs.

### 5.6. Nutritional Considerations Across the Menstrual Cycle and HC Use

Current sports nutrition guidelines increasingly recognize the relevance of hormone-informed approaches in female athletes, although evidence supporting rigid phase-based prescriptions remains limited [91]. Overall, nutritional strategies should prioritize adequate energy availability, carbohydrate support aligned with training demands, sufficient protein intake to optimize recovery, and individualized management of hydration status, micronutrient needs, and symptom burden [Table 2].

During the follicular phase, some women report reduced spontaneous energy intake and lower perceived appetite. Nevertheless, adequate carbohydrate availability remains crucial to sustain training intensity and support glycogen-dependent exercise. Protein intake should remain aligned with current recommendations for athletic populations (approximately 1.4–2.2 g·kg^−1^·day^−1^), particularly in athletes exposed to high training loads.

During the luteal phase, a small increase in resting energy expenditure has been reported in some women, which may contribute to higher energy requirements and appetite stimulation. Nutritional strategies during this phase may therefore benefit from closer attention to total energy intake, carbohydrate distribution, and meal timing, particularly in athletes reporting increased fatigue, cravings, or glycemic instability. Although protein requirements do not appear to differ meaningfully across menstrual phases, ensuring adequate intake and appropriate distribution across meals remains important to support recovery and preserve lean mass.

In athletes using HCs, chronic exposure to exogenous estrogens and progestins results in a more stable endocrine profile compared with the eumenorrheic cycle; however, this does not necessarily translate into stable metabolic responses. Nutritional strategies should therefore remain individualized, with particular attention to glucose regulation, appetite fluctuations, gastrointestinal tolerance, and perceived fatigue.

During the pill-free or placebo week, synthetic hormone concentrations decline, and withdrawal bleeding occurs. However, this interval does not replicate the endocrine physiology of the early follicular phase. Consequently, nutritional-related adjustments should not be automatically based on menstruation-oriented strategies but instead guided by monitoring of training tolerance, perceived exertion, and recovery status, particularly during periods of high training volume or reduced energy availability.

## 6. Symptoms as Functional Drivers of Performance

Menstrual cycle-related symptoms represent a key determinant of day-to-day performance and training tolerance in physically active or athletic women. While endocrine fluctuations define the underlying physiological context, symptom expression constitutes a more proximal and functionally relevant pathway through which this context translates into practical constraints on exercise capacity, perceived exertion, recovery, and training availability.

### 6.1. Prevalence and Heterogeneity of Symptoms

A high prevalence of menstrual cycle-related symptoms has been reported among physically active women and athletes, with estimates commonly exceeding 70–80% across sporting populations, primarily based on large-scale self-reported questionnaires and survey-based studies in athletic cohorts [116]. Frequently reported symptoms include dysmenorrhea, headache, bloating, gastrointestinal discomfort, sleep disturbances, mood changes, and increased fatigue. Importantly, symptom profiles and severity vary substantially across individuals and between cycles, even among women with comparable endocrine patterns. HC use may reduce symptom burden in some athletes and is often used strategically to manage menstrual-related discomfort or to manipulate bleeding timing around competitions [117].

### 6.2. Symptoms Versus Hormonal Phase: Implications for Training Tolerance and Performance

Accumulating evidence suggests that symptom burden may exert a stronger influence on perceived readiness and training quality than the menstrual cycle phase itself. Survey-based and observational studies indicate that pain, fatigue, gastrointestinal discomfort, and sleep disruption are frequently associated with reduced training output, missed sessions, and perceived performance impairments [14]. These findings provide a plausible explanation for why phase-based performance effects are often small or inconsistent at the group level despite biologically plausible endocrine mechanisms.

In applied settings, athletes often report that performance limitations coincide with periods of heightened symptoms rather than with a specific endocrine phase. Symptoms may also impair concentration and cognitive-motor control, potentially influencing movement quality and decision-making during complex sport-specific tasks [118]. Together, these findings support the view that symptoms act as functional mediators between endocrine context and real-world performance outcomes.

HC use may modulate symptom expression. In a large cohort of Norwegian athletes, HC users reported lower cycle-related symptom burden compared with non-users [115]. However, discontinuation rates due to adverse effects highlight substantial interindividual variability and the need for individualized symptom monitoring rather than assumptions of hormonal “stabilization”.

From a nutritional point of view, low vitamin D levels may contribute to the development or worsening of premenstrual symptoms, particularly those related to mood and inflammation [90,119]. A supplementation dose of approximately 2000 IU/day has been suggested as adequate for active individuals to help support symptom management [90,110]. This effect may be explained by vitamin D’s ability to reduce prostaglandin production and modulate inflammatory pathways, mechanisms that are involved in the pathophysiology of PMS and dysmenorrhea [90].

Zinc supplementation has also been investigated in PMS management. Clinical trials using 30 mg/day zinc gluconate for 12 weeks reported reductions in both physical and psychological PMS symptoms [120], while 50 mg/day zinc sulfate has been associated with decreased pain intensity and duration [121]. These effects are likely mediated by anti-inflammatory and neurotrophic actions, including modulation of BDNF, regulation of COX-2, and reduction in inflammatory cytokines [90].

Curcumin (100 mg twice daily) administered for 10 days per cycle (7 days before and 3 during menstruation) across three cycles has also been shown to reduce overall PMS severity [122], likely through similar anti-inflammatory and neuromodulatory mechanisms [90]. For a more comprehensive discussion of nutritional strategies for PMS management, readers are referred to the review by Brown et al. [90].

### 6.3. Toward Symptom-Informed Strategies

Symptom expression reflects the integrated interaction between endocrine modulation, metabolic regulation, inflammation, and central processes affecting fatigue, sleep, appetite, and gastrointestinal function. From a practical standpoint, monitoring symptom patterns across cycles may provide actionable insights into training tolerance and recovery needs. Symptom-informed frameworks, therefore, offer a more athlete-centered approach than rigid phase-based prescriptions, allowing individualized adjustment of training load and nutritional strategies to support performance consistency [9,116].

## 7. Integrative Framework: From Endocrine Context to Performance

The evidence summarized in this review supports a conceptual shift in how the menstrual cycle and HC use are interpreted in relation to athletic performance. Rather than acting as direct and deterministic drivers, sex hormone fluctuations define an endocrine context within which individual responses emerge through interactions with metabolic regulation, neuromuscular function, connective tissue behavior, and central mechanisms.

Importantly, average performance changes across menstrual phases or HC status appear modest and inconsistent, whereas interindividual variability is substantial. Factors such as training status, energy availability, nutritional habits, recovery capacity, and psychosocial stress influence how endocrine modulation translates into functional outcomes.

**Figure 3:**
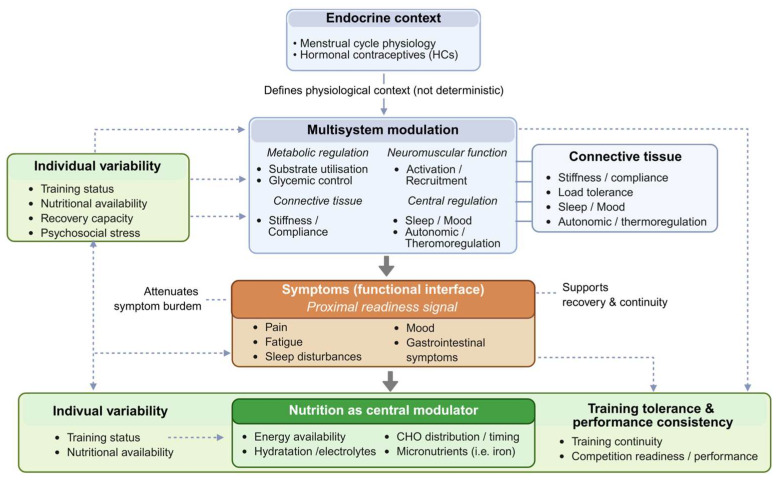
Integrative framework linking endocrine context, individual variability, symptom expression, and nutrition to performance outcomes in female athletes.

Within this framework, symptoms represent the most functionally relevant interface between endocrine physiology and real-world performance. Pain, fatigue, sleep disruption, gastrointestinal discomfort, and perceived energy availability integrate endocrine, metabolic, and central signals into tangible constraints on training tolerance, movement quality, and recovery. Therefore, performance readiness in female athletes is best understood as the product of dynamic interactions between endocrine context, individual variability, symptom expression, and behavioral regulation, with nutrition acting as a key modulator of these relationships. A schematic overview of this framework is provided in Figure 3.

## 8. Practical Implications for Sports and Health Professionals

Given the limited predictive value of menstrual cycle phase or HC status alone, applied decision-making should prioritize functional readiness and symptom burden, supported by simple monitoring strategies integrating training load, sleep quality, nutrition, and perceived recovery. Examples include menstrual tracking applications, athlete wellness questionnaires, daily symptom logs, and simple symptom scoring systems integrated into routine athlete monitoring platforms.

From a practical standpoint, nutrition should focus on maintaining adequate energy availability, supporting carbohydrate needs during demanding training blocks or periods of fatigue, ensuring sufficient protein intake for recovery, and managing hydration, electrolytes, and gastrointestinal tolerance based on individual responses. Integrating nutrition planning with symptom monitoring may allow practitioners to adjust fueling strategies during periods of increased fatigue, gastrointestinal discomfort, appetite changes, or sleep disturbances.

Athletes using HCs should be considered a distinct physiological population. HC use does not necessarily “stabilize” metabolic or performance-related outcomes, and individualized monitoring remains essential, particularly for glucose regulation, appetite patterns, fatigue perception, and gastrointestinal well-being.

Finally, reducing stigma around menstrual health and encouraging open communication between athletes and practitioners is critical for implementing effective, individualized strategies.

## 9. Conclusions

In conclusion, endogenous hormonal fluctuations across the menstrual cycle and exogenous hormonal modulation through HC use influence multiple physiological systems relevant to athletic performance. However, their direct effects on performance outcomes are generally modest and highly variable, with the available literature more often supporting small, context-dependent changes in selected physiological and symptoms-driven outcomes rather than larger or consistent effects on performance, limiting the practical utility of rigid phase-based models.

Although the present narrative review provides an integrative synthesis of current evidence, several limitations should be acknowledged. The narrative design does not follow a formal systematic screening process and therefore may be subject to potential selection bias. In addition, the available literature is characterized by considerable methodological heterogeneity, particularly regarding menstrual cycle phase verification (e.g., gold-standard hormonal verification methods vs. calendar-based or self-reported cycle tracking), HC formulations, and the assessment of performance-related outcomes. This heterogeneity also limits the possibility of drawing conclusions based on uniform effect sizes and supports a more cautious interpretation of the quantitative evidence currently available. Nevertheless, a key strength of this review lies in its integrative perspective, which connects endocrine physiology, metabolic regulation, symptom expression, and nutritional modulation within a unified conceptual framework relevant for physically active and athletic women.

Across both endocrine contexts, symptom expression emerges as an important mediator of day-to-day performance consistency and training tolerance within the multifactorial context of athletic performance, strongly shaped by metabolic regulation, nutritional status, training load, and recovery capacity. In this context nutritional considerations should not be limited to macronutrients supports alone, but should also include selected micronutrients with recognized relevance for female athletes, particularly iron, whose deficiency is reported to be substantially more prevalent in female than male athletes, as well as vitamin D, calcium, and vitamin B6 given their potential implications for fatigue, mood, bone health, and recovery. Therefore, athlete-centered approaches integrating symptom monitoring with individualized nutritional and training strategies provide the most practical and evidence-informed framework to support performance and long-term health in physically active and athletic women. Future research should prioritize well-controlled longitudinal designs that integrate hormonal verification, symptom tracking, and ecologically valid performance outcomes to further clarify the interactions between endocrine physiology, metabolism, and training adaptation.

## Figures and Tables

**Table 1:** Endocrine context in women: hormonal profiles and key physiological implications relevant to performance and nutrition.

Endocrine Context	Estradiol	Progesterone	Endocrine Pattern	Key Physiological Implications	Practical Interpretation
Natural (eumenorrheic) menstrual cycle—EF	↓	↓	Low-hormone state.	Higher likelihood of symptoms (e.g., pain, fatigue) in susceptible individuals; variable training tolerance.	Phase relevance is largely symptom-driven rather than hormone-driven.
Natural (eumenorrheic) menstrual cycle—LF/OV	↑↑ (peak)	↓	Estradiol-dominant.	Potential modulation of lipid oxidation and neuromuscular excitability; possible reduction in connective tissue stiffness.	Some athletes may experience favorable readiness, but responses are inconsistent.
Natural (eumenorrheic) menstrual cycle—ML	↑	↑↑	Progesterone-dominant.	Increased basal temperature and ventilatory drive; possible alterations in insulin sensitivity, appetite, fluid balance, and fatigue perception.	May increase nutrition-related demands (carbohydrate handling, hydration, recovery support).
Natural (eumenorrheic) menstrual cycle—LT/premenstrual	↓	↓	Hormonal withdrawal.	Greater symptom burden in women with PMS (mood changes, cravings, GI discomfort, sleep disturbances); inflammation-related cravings.	Practical relevance depends strongly on PMS severity and individual symptom patterns.
HC use	↓ endogenous	↓ endogenous	Suppressed ovulation; chronic exposure to synthetic hormones.	Reduced cyclic hormonal variation but heterogeneous metabolic and symptom responses; endocrine profile does not replicate any natural cycle phase.	HCs should not be assumed to “stabilize” physiology; individualized monitoring remains essential.

**Abbreviations:** EF, early follicular; LF, late follicular; OV, ovulatory; ML, mid-luteal; LT, late luteal; HC, hormonal contraceptive; PMS, premenstrual syndrome; GI, gastrointestinal. **Note:** Physiological responses to HC use may vary according to formulation, delivery route, and individual sensitivity. **Symbols:** ↑ increase; ↓ decrease. Arrows represent the potential direction of endocrine modulation and should not be interpreted as deterministic effects.

**Table 2:** Summary of nutritional considerations across the menstrual cycle and hormonal contraceptive use in female athletes.

Domain	Natural (Eumenorrheic) Menstrual Cycle	Hormonal Contraceptive Use
Energy availability/intake	Appetite and intake may increase during the luteal phase by 100–300 kcal/day, particularly in women with PMS; risk of mismatched fueling across phases. Target: >45 kcal/kg FFM/day.	Energy intake may appear more stable, but appetite, fatigue, and tolerance remain highly individualized. Target: >45 kcal/kg FFM/day
Carbohydrate metabolism	Estradiol may favor lipid oxidation; progesterone may contribute to altered glycemic regulation during the luteal phase. Practical relevance is context-dependent. Post-exercise: ~1.2 g/kg rapid carbohydrates. During exercise: 30–60 g/h for sessions > 60 min to compensate for reduced gluconeogenesis in the luteal phase.	Some evidence suggests altered glucose handling in some users; effects are heterogeneous and formulation-dependent. Prioritize low-glycemic index carbohydrates during active pill weeks to mitigate transient insulin resistance.
Protein metabolism/recovery	Recent tracer studies suggest no meaningful phase-dependent differences in MPS or proteolysis; recovery capacity may still vary due to symptoms and training load. Target: 1.4–2.2 g/kg/day and post-exercise protein intake (target ~0.32–0.38 g/kg).	HC phase (active vs. pill-free) does not appear to systematically alter anabolic responsiveness; no clear rationale for pill-week protein periodization. Target: 1.4–2.2 g/kg/day and post-exercise protein intake (target ~0.32–0.38 g/kg).
Inflammation/symptoms	Luteal-phase increases in inflammation-related symptoms (fatigue, cravings, discomfort) may indirectly affect training tolerance and recovery nutrition. Vitamin D (2000 IU/day), zinc (30–50 mg/day), and curcumin (100 mg twice daily) may reduce symptom severity.	HC use may reduce symptom severity in some women, but individual responses remain variable.
Fluid balance/ hydration	Progesterone-related fluid retention may contribute to transient body mass fluctuations and perceived bloating.	Fluid shifts and bloating may occur depending on individual response and formulation; monitoring remains essential.
GI tolerance	GI symptoms (bloating, bowel changes) may fluctuate across the cycle and affect feeding strategies around exercise.	GI tolerance may improve or worsen depending on individual response; symptom monitoring is recommended.
Iron status	Menstrual blood loss and hepcidin modulation may increase risk of iron deficiency; absorption may be more favorable in early follicular phase. Estimated requirement in endurance athletes: ~30 mg/day.	Reduced bleeding may lower risk of deficiency; supplementation should be based on biomarkers rather than routine use. Supplement only when deficiency is confirmed. Some oral contraceptives provide 25 mg iron during placebo days.
Overall practical implication	Nutritional planning should prioritize training load, energy availability, and symptom expression rather than phase-based assumptions.	HC use does not guarantee metabolic “stabilization”; individualized monitoring remains the most appropriate approach.

**Abbreviations:** HC, hormonal contraceptive; PMS, premenstrual syndrome; MPS, muscle protein synthesis; GI, gastrointestinal.

## Data Availability

No new data were created or analyzed in this study.
